# Acute Myocardial Injury in Patients Presenting With Upper Gastrointestinal Bleeding: Prevalence, Predictors, Troponin Dynamics, and In-Hospital Outcomes

**DOI:** 10.7759/cureus.119221

**Published:** 2026-11-06

**Authors:** Nayyar Abbas, Fatima N Shahid, Rameesha Amjad, Muhammad Waleed Amjad, Samia Samar Arooj, Saadia Sehrish, Aamir Bashir, Mehak Yusuf, Aymen Shafiq, Sharjeel Mahmood

**Affiliations:** 1 General Internal Medicine, Oxford University Hospitals NHS Foundation Trust, Oxford, GBR; 2 Gastroenterology and Hepatology, Pakistan Kidney and Liver Institute and Research Center, Rawalpindi, PAK; 3 Acute Medicine, Royal Albert Edward Infirmary, Wigan, GBR; 4 Cardiac Surgery, Ghurki Trust Teaching Hospital, Lahore, PAK; 5 Medicine, Punjab Rangers Teaching Hospital, Lahore, PAK; 6 Anesthesia and Critical Care, Shalamar Hospital, Lahore, PAK; 7 Public Health, University of Lahore, Lahore, PAK; 8 General Surgery, Mayo Hospital, Lahore, PAK

**Keywords:** acute myocardial injury, cardiac troponin, clinical predictors, high-sensitivity troponin, in-hospital mortality, myocardial ischemia, risk stratification, troponin dynamics, type 2 myocardial infarction, upper gastrointestinal bleeding

## Abstract

Background: Upper gastrointestinal bleeding (UGIB) may cause anemia, hypovolemia, and hemodynamic compromise, potentially contributing to myocardial injury during the acute illness. Troponin elevation in this setting requires careful interpretation because an isolated measurement cannot reliably distinguish acute injury from elevation without qualifying change or establish myocardial infarction. The frequency, temporal evolution, and clinical significance of acute myocardial injury in patients with UGIB remain incompletely characterized. This prospective multicenter study evaluated its prevalence, associated admission factors, serial troponin dynamics, and relationship with in-hospital outcomes, including subsequent mortality among patients surviving the first 24 hours.

Methods: This prospective multicenter observational cohort study enrolled 1,000 adults at tertiary care hospitals in Punjab, Pakistan, between January 2025 and August 2026. Cardiac troponin I measurements were scheduled at presentation, 3-6 hours, and 20-24 hours. Acute injury required a concentration above the applicable 99th-percentile reference limit and a serial maximum-minimum range of ≥10 ng/L and ≥20% of the earliest observed concentration, using at least two measurements spanning ≥3 hours. Prevalence was estimated with Wilson confidence intervals (CIs). Multivariable logistic regression examined admission factors associated with injury. A 24-hour landmark analysis evaluated subsequent mortality among patients alive and still hospitalized with classifiable injury status.

Results: Injury status was classifiable in 960 patients; 104 had acute injury, giving a prevalence of 10.8% (95% CI: 9.0-13.0%). A further 131 patients (13.6% of classifiable assessments) had troponin elevation without qualifying change, while 40 assessments were indeterminate. Older age (adjusted odds ratio (aOR) per decade: 1.40; 95% CI: 1.21-1.63), coronary artery disease (aOR: 1.82; 95% CI: 1.06-3.10), and lower hemoglobin (aOR per 1 g/dL decrease: 1.27; 95% CI: 1.06-1.51) were associated with acute injury. Fourteen acute-injury cases (13.5%) first met the classification criterion at the late measurement. Among 810 landmark patients with known outcomes, 34 died. Subsequent mortality was 15.2% with acute injury versus 2.8% without acute injury (aOR: 3.47; 95% CI: 1.58-7.62; p=0.002). An adverse differential assignment of unresolved mortality outcomes attenuated this association (aOR: 1.86; 95% CI: 0.94-3.69).

Conclusions: Acute myocardial injury affected approximately one in 10 classifiable patients with UGIB and was associated with older age, coronary artery disease, lower hemoglobin, and higher subsequent in-hospital mortality. Serial measurements distinguished acute changes from elevation without qualifying change. The mortality association requires cautious interpretation because of limited events, residual confounding, and incomplete follow-up.

## Introduction

Acute upper gastrointestinal bleeding (UGIB) is a common reason for emergency admission and may cause substantial blood loss, circulatory compromise, and organ dysfunction. Initial assessment focuses on resuscitation, assessment of bleeding severity, and identification of patients requiring endoscopic intervention [1]. Although control of hemorrhage is essential, concurrent cardiovascular disease and cardiac complications can influence the clinical course. The 2026 European Society of Gastrointestinal Endoscopy guideline emphasizes assessment and stabilization before appropriately timed treatment of peptic ulcer bleeding, one of the major causes of UGIB [2].

Hemorrhage may reduce myocardial oxygen delivery through anemia and hypovolemia, while tachycardia and physiological stress increase oxygen demand. Cardiac troponin can therefore become elevated during UGIB even when the presentation is dominated by bleeding. Interpretation requires distinction between acute injury, persistent troponin elevation, and infarction. The Fifth Universal Definition of Myocardial Infarction, published in 2026, supports sex-specific reference limits and separates myocardial injury from infarction, with the latter requiring evidence of ischemia [3].

Published estimates vary with patient selection and laboratory methods. Iser et al. prospectively studied 156 patients with UGIB using serial conventional troponin I measurements at presentation, 12 hours, and 24 hours; 30 patients (19%) exceeded the study threshold [4]. Iqbal et al. reported troponin elevation in 10% of 290 patients with acute gastrointestinal bleeding who underwent testing, with higher 30-day mortality among those with elevated concentrations [5]. Kousa et al. found abnormal troponin in 17% of 172 tested patients with acute gastrointestinal bleeding, with longer hospitalization and greater cardiac investigation but no significant mortality difference [6]. The latter two studies included gastrointestinal bleeding more broadly and do not provide directly comparable UGIB-only estimates.

Previous studies have differed in patient selection, assay thresholds, and sampling schedules [4-7]. Although serial troponin testing has been reported, explicit separation of qualifying dynamic injury from elevation without qualifying change, together with temporally defined mortality analyses, remains limited. The present study therefore examined these biochemical patterns during the first 24 hours of UGIB and evaluated their association with subsequent in-hospital outcomes.

To address these gaps, this multicenter prospective observational study aimed to determine the prevalence of acute myocardial injury within the first 24 hours of presentation using standardized serial high-sensitivity cardiac troponin measurements. Secondary objectives were to identify admission factors associated with myocardial injury; characterize troponin dynamics, including absolute and relative changes and time to observed peak; and compare in-hospital mortality, intensive care admission, rebleeding, transfusion requirements, time to endoscopy, and length of stay according to injury status. Additionally, the study evaluated the association between early myocardial injury and subsequent in-hospital mortality using a 24-hour landmark analysis and described myocardial injury phenotypes and evidence supporting an ischemic mechanism.

## Materials and methods

Study design and setting

This multicenter prospective observational cohort study was conducted at two tertiary care hospitals in Punjab, Pakistan: Gujranwala Teaching Hospital, Gujranwala, and Shalamar Hospital, Lahore. Recruitment was conducted from January 2025 to August 2026. Consecutive adult patients admitted through emergency, medical, gastroenterology, or surgical services with acute UGIB were screened for eligibility. The study investigated the prevalence, clinical predictors, troponin dynamics, and in-hospital outcomes associated with acute myocardial injury. Clinical management remained under the discretion of treating physicians without study-mandated therapeutic interventions. Reporting was guided by the Strengthening the Reporting of Observational Studies in Epidemiology (STROBE) statement [8].

Study population and sample size

Patients aged 18 years or older presenting with clinically suspected acute UGIB requiring hospitalization were eligible. Only the first eligible admission per patient was included. Exclusion criteria comprised a subsequently confirmed isolated lower gastrointestinal or non-gastrointestinal bleeding source, bleeding developing during hospitalization for another primary condition, repeat admissions, and refusal of consent where required. Patients with pre-existing cardiovascular disease or chronic kidney disease were not excluded. Incomplete serial sampling, early deterioration, or death did not result in retrospective exclusion, with insufficiently assessed cases classified as indeterminate.

Consecutive nonprobability sampling was employed, whereby patients presenting sequentially to the participating hospitals during the study period were screened and enrolled if they fulfilled the predefined eligibility criteria and provided consent where required. Unlike probability sampling, participants were not selected through randomization; instead, eligible patients were recruited as they presented. This approach was chosen because acute UGIB is an emergency presentation with unpredictable admission patterns, making random sampling operationally challenging. Consecutive recruitment was intended to minimize investigator-driven selection bias, capture a broad spectrum of disease severity, and reflect routine clinical practice across both participating centers. Nevertheless, as a nonprobability sampling method, it does not eliminate selection bias or guarantee representativeness of the wider population. Sample size was estimated using the single-proportion formula, $\mathrm{n}=\mathrm{Z}^{&sup2;}\mathrm{p}(1-\mathrm{p})/\mathrm{d}^{2}$, assuming a conservative prevalence of 50%, a 95% confidence level, and a 3.5% absolute precision. The minimum required evaluable sample was 784 patients. Allowing for 10% unclassifiable assessments increased the recruitment requirement to 872 patients, while a target of 1,000 participants was established to improve precision and support secondary analyses. This calculation was intended to ensure precision of the prevalence estimate rather than adequate power for every adjusted association.

Data collection and baseline assessment

Trained research personnel collected data using standardized case-record forms and assigned unique study identifiers. Baseline variables included age, sex, presenting symptoms and duration, previous UGIB, coronary artery disease, heart failure, chronic kidney disease, diabetes mellitus, hypertension, liver disease, and pre-admission antiplatelet, anticoagulant, and nonsteroidal anti-inflammatory drug use. Initial physiological measurements included systolic blood pressure, heart rate, respiratory rate, oxygen saturation, and consciousness level. Laboratory investigations included hemoglobin, platelet count, serum urea, creatinine, international normalized ratio, albumin, and lactate where available. Pretreatment measurements were preferentially recorded, with preceding resuscitative interventions documented. Collection times and major clinical interventions were documented to establish temporal relationships.

The shock index was calculated by dividing the presentation heart rate (beats/min) by the systolic blood pressure (mmHg). Higher values indicate greater tachycardia relative to systolic pressure and potentially greater circulatory compromise. The index was summarized as a continuous measure in this study.

The Glasgow-Blatchford score (GBS) was calculated from presentation blood urea, sex-specific hemoglobin, systolic blood pressure, pulse, melena, syncope, hepatic disease, and heart failure using the original weighted scoring system [9]. Urea concentrations of 6.5 to <8.0, 8.0 to <10.0, 10.0 to 25.0, and >25.0 mmol/L scored 2, 3, 4, and 6 points, respectively. In males, hemoglobin concentrations of 12.0 to <13.0, 10.0 to <12.0, and <10.0 g/dL scored 1, 3, and 6 points; in females, concentrations of 10.0 to <12.0 and <10.0 g/dL scored 1 and 6 points. Systolic pressures of 100 to <110, 90 to <100, and <90 mmHg scored 1, 2, and 3 points. Pulse ≥100 beats/min and melena each scored 1 point; syncope, hepatic disease, and heart failure each scored 2 points. Other values or absent features scored 0. Points were summed to give a total of 0 to 23, with higher scores indicating a greater likelihood of requiring hospital-based intervention. Scores of 0 to 1 identify a very-low-risk group in clinical guidance [1]. GBS was retained as a numerical score in the analyses. The GBS was selected because it is an established, readily applicable pre-endoscopic risk stratification tool for patients presenting with UGIB. By integrating clinical features, hemodynamic parameters, and laboratory measurements available at presentation, it provides a standardized assessment of baseline UGIB-related clinical risk. In this study, the score was used to compare baseline risk between myocardial injury groups and was included as an adjustment variable in the 24-hour landmark mortality analysis to account for differences in initial UGIB-related clinical risk. The GBS was not interpreted as a direct measure of blood-loss volume or as a diagnostic tool for myocardial injury.

The Glasgow Coma Scale (GCS) was assessed by summing the best eye-opening, verbal, and motor responses. Eye opening was scored from 1 (none) to 4 (spontaneous), verbal response from 1 (none) to 5 (oriented), and motor response from 1 (none) to 6 (obeys commands). Intermediate eye scores were 2 for opening to pressure and 3 for opening to sound. Intermediate verbal scores were 2 for sounds, 3 for words, and 4 for confused conversation. Intermediate motor scores were 2 for extension, 3 for abnormal flexion, 4 for normal flexion or withdrawal, and 5 for localization. Total scores ranged from 3 to 15; a score of 15 indicated full responsiveness on the scale, while lower scores indicated increasing impairment of consciousness. GCS was summarized numerically without assigning traumatic-brain-injury severity categories.

Troponin measurement and myocardial injury classification

High-sensitivity cardiac troponin I (hs-cTnI) was measured using the Abbott Diagnostics Alinity i platform (Abbott Park, Illinois, United States) at both participating institutions. The same assay was used throughout the study. The sex-specific upper reference limits applied in the study were 16 ng/L for females and 34 ng/L for males, reflecting the locally used laboratory thresholds. Serial troponin testing was implemented as part of routine ward care. Three measurements were scheduled: at presentation, preferably within 60 minutes; 3-6 hours after the initial measurement; and 20-24 hours after arrival. Actual collection times were documented. Additional testing was performed when clinically indicated, but the primary injury-assessment window remained restricted to the first 24 hours.

Before examination of the study results, acute myocardial injury was operationally defined as at least one concentration above the applicable reference limit, together with an observed maximum-minimum difference of ≥10 ng/L and ≥20% of the earliest observed concentration, using at least two measurements spanning ≥3 hours. This combined criterion required both an absolute and a proportional change: a large percentage change at a low initial concentration could not qualify solely on that basis, while a small proportional fluctuation at a high initial concentration could not qualify solely because its absolute magnitude exceeded 10 ng/L. Contemporary guidance supports considering absolute and relative changes in relation to assay characteristics and baseline concentrations [3]. However, the specific combined rule used here was a prespecified study definition rather than a validated diagnostic threshold for UGIB.

Patients with at least one elevated concentration but without a qualifying dynamic change were classified as having elevation without qualifying change. Patients whose available serial concentrations did not exceed the reference limit were classified as having no biochemical injury detected. These categories describe the observed measurements and do not establish chronic injury or exclude injury developing after the final available sample. Assessments with fewer than two interpretable measurements spanning ≥3 hours were classified as indeterminate.

Biochemical injury categories were defined by the serial troponin criteria described above. Recorded ischemic symptoms, electrocardiographic findings, and available cardiac imaging or coronary findings were summarized descriptively to provide clinical context. These descriptive findings were not used to estimate myocardial infarction prevalence or the frequency of individual infarction subtypes. Acute myocardial injury was distinguished conceptually from myocardial infarction, which requires additional evidence supporting an ischemic mechanism [10,11].

Clinical outcomes and follow-up

Patients were followed throughout their index hospitalization until discharge, death, or transfer, with subsequent hospital outcomes obtained where available. Daily assessments documented endoscopic findings, hemostatic interventions, packed red-cell transfusion requirements, vasopressor administration, intensive care unit (ICU) admission, clinically significant rebleeding, rescue embolization or surgery, and vital status. Clinically significant rebleeding was assessed using documented recurrent overt bleeding after initial stabilization, accompanied by hemodynamic deterioration, a clinically important hemoglobin decline, or repeat hemostatic intervention.

The primary endpoint was the prevalence of acute myocardial injury detected within 24 hours among patients with classifiable assessments. The principal clinical outcome was all-cause in-hospital mortality. Secondary outcomes included ICU admission, clinically significant rebleeding, total packed red-cell units transfused, time from presentation to endoscopy, and hospital length of stay.

A prespecified 24-hour landmark analysis was performed to evaluate the association between early myocardial injury and subsequent in-hospital mortality while establishing a clear temporal relationship between exposure classification and outcome assessment. Because acute myocardial injury was determined using serial troponin measurements obtained during the first 24 hours of admission, analyzing mortality from the time of presentation could introduce immortal-time bias and temporal ambiguity, particularly when deaths occurred before myocardial injury status could be established. The 24-hour landmark approach ensured that injury status was determined before the mortality observation period began, thereby reducing the risk of attributing early deaths to an exposure that had not yet been fully ascertained. However, this approach necessarily excluded patients who died or otherwise exited hospital care before the landmark and therefore evaluated mortality conditional on survival and continued hospitalization at 24 hours. This analysis included patients alive and still hospitalized at 24 hours with classifiable injury status. Deaths, discharges, and transfers preceding the landmark were described separately. Full-admission outcomes were additionally summarized descriptively according to myocardial injury status.

Statistical analysis

Data were analyzed using Python (version 3.14) (Python Software Foundation, Wilmington, Delaware, United States). The distributions of continuous variables were assessed using histograms, normal quantile-quantile (Q-Q) plots, and the Shapiro-Wilk test. Normally distributed variables were expressed as mean±standard deviation, while skewed variables were presented as median and interquartile range. Categorical variables were summarized as frequencies and percentages using available denominators.

Acute myocardial injury prevalence was estimated among classifiable patients with 95% Wilson confidence intervals (CIs). Baseline characteristics and clinical outcomes were compared between patients with and without acute myocardial injury. Troponin measurements were additionally summarized across the four biochemical assessment categories. For unadjusted comparisons of continuous variables, Welch's t-test was used for variables assessed as approximately normally distributed, while the Mann-Whitney U test was applied to variables with non-normal distributions. Categorical variables were compared using Pearson's chi-squared test, with Fisher's exact test applied where appropriate. The statistical tests performed for individual comparisons, along with their corresponding test statistics and p-values, are reported in the relevant tables.

Multivariable logistic regression was employed to identify admission factors independently associated with acute myocardial injury among patients with classifiable assessments. This approach was considered appropriate because acute myocardial injury was a binary outcome, and several potential predictors, including age, pre-existing cardiovascular disease, renal dysfunction, anemia, and hemodynamic parameters, may be interrelated. Although unadjusted analyses identified differences between patients with and without acute myocardial injury, these comparisons could not adequately account for potential confounding. Multivariable logistic regression allowed simultaneous adjustment for clinically relevant demographic, cardiovascular, hematological, and physiological variables, as well as differences between participating centers, thereby providing adjusted estimates of the associations between individual admission factors and myocardial injury. The analysis was intended to identify independent statistical associations rather than establish causal relationships or develop a validated prediction model. The adjusted model included age, sex, coronary artery disease, chronic kidney disease, hemoglobin, systolic blood pressure, heart rate, and study center, with center entered as a fixed effect. Continuous predictors were retained without dichotomization. Odds ratios were expressed per 10-year increase in age, 1 g/dL decrease in hemoglobin, 10 mmHg decrease in systolic pressure, and 10 beats/min increase in heart rate. Adjusted odds ratios (aORs) with 95% Wald CIs were reported. Collinearity was assessed using variance inflation factors. Exploratory restricted cubic spline models were compared with the linear model using likelihood-ratio tests to assess possible nonlinearity.

Troponin dynamics were characterized using admission concentrations, absolute and relative changes, observed peak concentrations, peak-to-reference-limit ratios, and time to observed peak. Complete and incomplete sampling trajectories were distinguished. Full-admission outcomes were summarized using absolute risks and effect estimates with 95% CIs where appropriate.

The association between acute myocardial injury and subsequent in-hospital mortality was examined using logistic regression among patients alive and still hospitalized at 24 hours with classifiable injury status and known subsequent outcomes. The primary model included injury status, age, GBS, and chronic kidney disease. Sensitivity analyses omitted chronic kidney disease, added study center, restricted the population to patients completing all three scheduled troponin measurements, or excluded elevation without qualifying change from the comparator. Additional analyses examined extreme assignments of unresolved mortality outcomes. A Firth bias-reduced estimate was calculated as a sensitivity point estimate; an accompanying profile-likelihood confidence interval was not available. These analyses assessed the consistency of the observed association rather than establishing a validated prognostic model.

Missing values were quantified for individual variables and troponin measurements. Missing troponin values were not imputed, and indeterminate assessments were not considered negative. Sensitivity analyses included restriction to patients completing all three scheduled measurements and stratification by chronic kidney disease and variceal versus nonvariceal bleeding. Prevalence bounds were examined by alternatively assigning indeterminate assessments to injury and noninjury categories. Missing mortality outcomes were reported separately without assuming survival. Two-sided p-values below 0.05 were considered statistically significant. P-values for secondary outcome comparisons were not adjusted for multiple testing and were interpreted as exploratory, with emphasis on effect sizes and confidence intervals.

Covariates in the multivariable myocardial injury model were selected on clinical grounds to account for demographic characteristics, pre-existing cardiovascular and renal disease, anemia, hemodynamic status, and potential center-related differences. The landmark mortality model incorporated age, GBS, and chronic kidney disease to adjust for important baseline clinical characteristics while limiting model complexity given the relatively small number of mortality events. All covariates included in the principal myocardial injury model were complete among the 960 classifiable patients, and no imputation was required. Variables with incomplete observations that were not included in this model, including BMI, lactate, and albumin, were analyzed using available-case denominators. Missing troponin measurements were not imputed, and patients with insufficient measurements for injury classification were categorized as indeterminate.

Ethical considerations and data management

Ethical approval was obtained from the Institutional Review Board of Shalamar Medical and Dental College (approval number: 0938). Informed consent was obtained from participants. Serial troponin measurements were implemented as part of routine ward care. Participation did not delay emergency resuscitation, blood transfusion, endoscopic intervention, or other clinically indicated treatment. Abnormal findings were communicated to the treating teams through routine clinical reporting pathways.

Patient information was anonymized using unique study identifiers, with identifying details stored separately from the analytical dataset. Electronic records were maintained on password-protected, institution-approved systems accessible only to authorized research personnel. Data completeness, laboratory values, measurement units, and collection timestamps were checked against source records before analysis. Confidentiality was maintained according to institutional ethical and data-protection requirements.

## Results

Study population and data completeness

The dataset comprised 1,000 unique patients, including 630 men (63%), with a median age of 58.0 years (IQR: 47.0-69.0). The recorded bleeding sources were peptic ulcer disease in 428 (42.8%), erosive disease in 153 (15.3%), variceal bleeding in 148 (14.8%), Mallory-Weiss lesions in 68 (6.8%), malignancy in 55 (5.5%), and other upper gastrointestinal sources in 54 (5.4%). The source remained unconfirmed in 94 patients (9.4%).

Of 3,000 scheduled troponin measurements, 2,644 (88.1%) were observed. Presentation, first-repeat, and late measurements were available in 962 (96.2%), 915 (91.5%), and 767 patients (76.7%), respectively. Actual median collection times were 0.4 h (IQR: 0.3-0.6), 4.7 h (IQR: 4.1-5.4), and 21.9 h (IQR: 20.9-23.0). All three measurements were available in 693 patients (69.3%); 267 (26.7%) had two, 31 (3.1%) had one, and nine (0.9%) had none. Missing samples reflected missed draws (n=159), laboratory or sample failures (n=62), discharge before the scheduled sample (n=115), death before sampling (n=15), or transfer before sampling (n=5).

Injury status was classifiable in 960 patients (96%) and indeterminate in 40 (4%). All variables used in the principal injury model were complete among classifiable patients. In the full cohort, BMI, lactate, and albumin were missing in 75 (7.5%), 166 (16.6%), and 64 patients (6.4%), respectively. Vital status at the end of hospitalization was known for 966 patients, among whom 49 deaths were recorded (5.1%). Thirty-four transfers had unresolved subsequent vital status and were excluded from mortality denominators. Analysis populations are shown in Figure 1.

**Figure 1:**
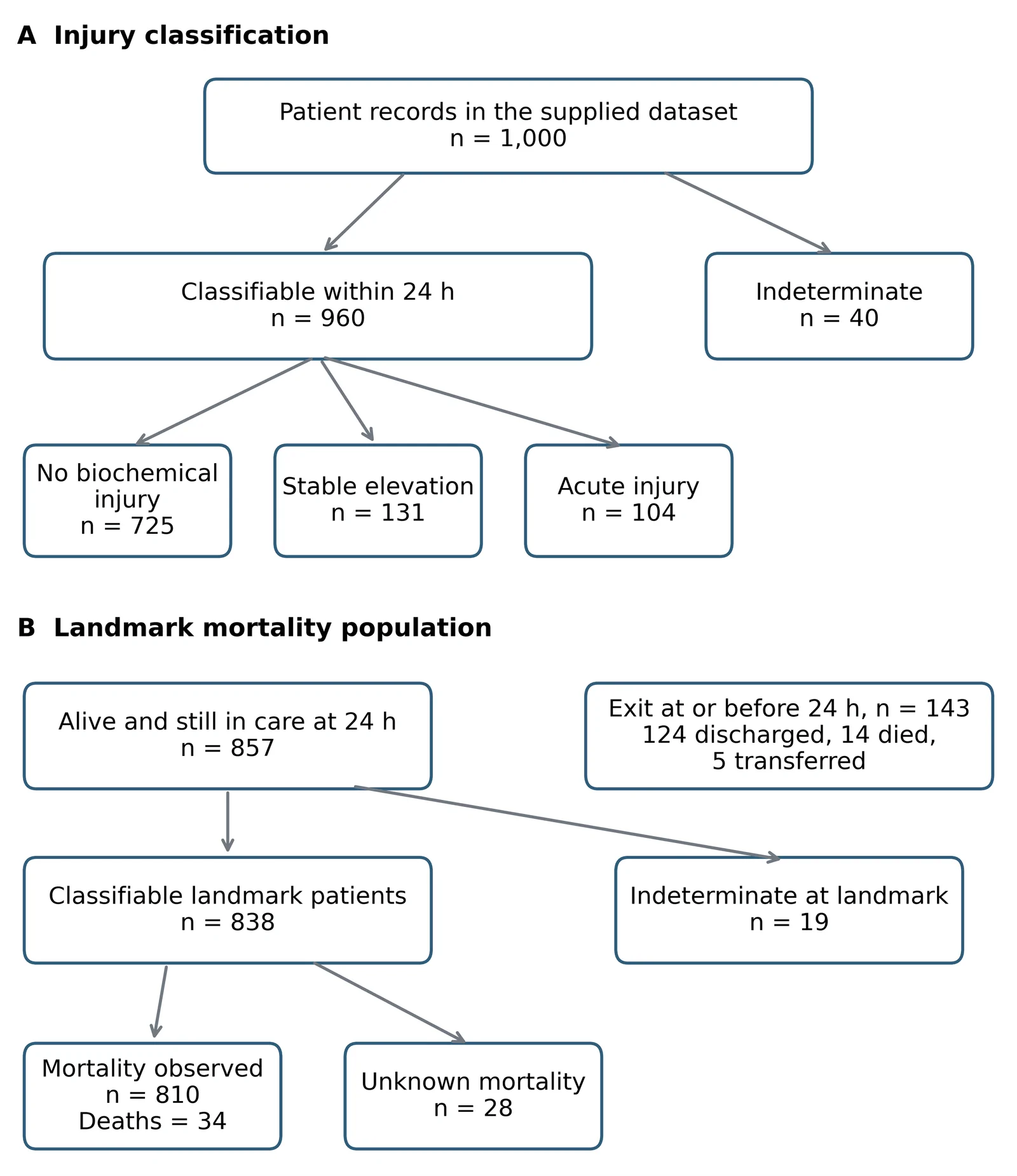
Analysis populations and landmark eligibility Panel A shows the biochemical classification of 1,000 enrolled patients: 960 had classifiable assessments, and 40 had indeterminate assessments. Classifiable patients comprised 104 with acute injury, 131 with elevation without qualifying change, and 725 with no biochemical injury detected. Panel B shows selection for the 24-hour landmark analysis. After 143 early exits, 857 patients remained in care; exclusion of 19 indeterminate assessments left 838 eligible patients. Subsequent mortality was known for 810 patients, among whom 34 deaths occurred, and unresolved for 28.

Prevalence and baseline characteristics

Acute myocardial injury was identified in 104 of 960 classifiable patients, corresponding to a prevalence of 10.8% (95% Wilson CI: 9.0-13.0%). A further 131 patients (13.6%) had troponin elevation without qualifying change, and 725 (75.5%) had no biochemical injury detected. The prespecified nonacute comparator therefore comprised 856 patients, including both elevation without qualifying change and no biochemical injury detected. Assigning all 40 indeterminate assessments to noninjury or acute injury yielded whole-cohort prevalence bounds of 10.4% and 14.4%, respectively; these are missing-status bounds rather than confidence limits.

Patients with acute injury were older than those without acute injury (median: 64.0 vs. 58.0 years; p<0.001) and more frequently had coronary artery disease (26% vs. 15.1%; p=0.004) or chronic kidney disease (23.1% vs. 12.3%; p=0.002). They had lower hemoglobin and systolic pressure, a higher heart rate, and greater bleeding severity as measured by the GBS (median: 12.0 vs. 8.0; p<0.001). Baseline characteristics, available denominators, test statistics, and p-values are presented in Table 1.

**Table 1:** Baseline characteristics by acute injury status Values are presented as mean±SD for BMI, systolic and diastolic blood pressure, and hemoglobin; median (IQR) for other continuous variables; and n/N (%) for categorical variables. Comparisons between the acute-injury and no-acute-injury groups used two-sided Welch t-tests for the four variables presented as mean±SD and two-sided Mann-Whitney U tests for the remaining continuous variables, with U reported for the acute-injury group. All categorical comparisons in this table used Pearson chi-squared tests without continuity correction, with 1 degree of freedom; no comparison used Fisher's exact test. The n values beside continuous summaries indicate the numbers of patients with available measurements in the corresponding columns. Lactate was available in 800 classifiable patients (92 with acute injury and 708 without acute injury) and albumin in 898 (97 and 801, respectively); missing measurements were excluded from the relevant summaries and comparisons. The no-acute-injury group includes elevation without qualifying change and no biochemical injury detected. Forty indeterminate assessments were excluded. Center 1 denotes Gujranwala Teaching Hospital, Gujranwala, Pakistan, and Center 2 denotes Shalamar Hospital, Lahore, Pakistan.

Characteristic	Classifiable cohort (n=960)	Acute injury (n=104)	No acute injury (n=856)	Test statistic	P-value
Age, years	58.0 (47.0-69.0)	64.0 (55.0-73.0)	58.0 (46.0-68.0)	U=56712.00	<0.001
Male sex	607/960 (63.2%)	74/104 (71.2%)	533/856 (62.3%)	χ²=3.15	0.076
Center 1	539/960 (56.1%)	57/104 (54.8%)	482/856 (56.3%)	χ²=0.08	0.771
Center 2	421/960 (43.9%)	47/104 (45.2%)	374/856 (43.7%)	χ²=0.08	0.771
BMI, kg/m²	25.8±4.2; n=888	25.8±4.6; n=95	25.8±4.2; n=793	t=0.01; df=113.1	0.995
Coronary artery disease	156/960 (16.2%)	27/104 (26%)	129/856 (15.1%)	χ²=8.08	0.004
Heart failure	74/960 (7.7%)	13/104 (12.5%)	61/856 (7.1%)	χ²=3.76	0.052
Chronic kidney disease	129/960 (13.4%)	24/104 (23.1%)	105/856 (12.3%)	χ²=9.32	0.002
Diabetes mellitus	188/960 (19.6%)	21/104 (20.2%)	167/856 (19.5%)	χ²=0.03	0.868
Hypertension	328/960 (34.2%)	36/104 (34.6%)	292/856 (34.1%)	χ²=0.01	0.919
Cirrhosis or liver disease	183/960 (19.1%)	23/104 (22.1%)	160/856 (18.7%)	χ²=0.70	0.401
Previous upper gastrointestinal bleeding	178/960 (18.5%)	22/104 (21.2%)	156/856 (18.2%)	χ²=0.53	0.468
Antiplatelet use	155/960 (16.1%)	21/104 (20.2%)	134/856 (15.7%)	χ²=1.41	0.235
Anticoagulant use	64/960 (6.7%)	11/104 (10.6%)	53/856 (6.2%)	χ²=2.87	0.090
Nonsteroidal anti-inflammatory drug use	185/960 (19.3%)	15/104 (14.4%)	170/856 (19.9%)	χ²=1.76	0.184
Hematemesis	593/960 (61.8%)	69/104 (66.3%)	524/856 (61.2%)	χ²=1.03	0.309
Melena	721/960 (75.1%)	81/104 (77.9%)	640/856 (74.8%)	χ²=0.48	0.487
Syncope	104/960 (10.8%)	16/104 (15.4%)	88/856 (10.3%)	χ²=2.50	0.114
Symptom duration, h	12.7 (7.0-22.2)	10.1 (6.5-17.5)	13.0 (7.1-22.7)	U=39243.50	0.048
Systolic blood pressure, mmHg	122.5±22.0	111.0±23.7	123.9±21.4	t=-5.26; df=124.2	<0.001
Diastolic blood pressure, mmHg	73.8±14.8	66.9±15.9	74.6±14.5	t=-4.71; df=124.6	<0.001
Heart rate, beats/min	89.0 (73.0-106.0)	104.5 (86.0-125.0)	88.0 (72.0-105.0)	U=60273.00	<0.001
Respiratory rate, breaths/min	18.0 (16.0-20.0)	19.0 (17.8-21.0)	18.0 (16.0-20.0)	U=53870.50	<0.001
Oxygen saturation, %	96.5 (95.1-97.8)	96.2 (94.6-97.6)	96.5 (95.2-97.8)	U=40148.00	0.102
Glasgow Coma Scale	15.0 (15.0-15.0)	15.0 (15.0-15.0)	15.0 (15.0-15.0)	U=40328.00	<0.001
Hemoglobin, g/dL	9.8±2.0	8.5±2.0	9.9±2.0	t=-7.05; df=129.2	<0.001
Platelets, ×10⁹/L	211.0 (163.0-257.0)	214.5 (150.5-262.0)	210.0 (164.8-256.0)	U=44432.50	0.976
Urea, mmol/L	7.9 (5.2-12.3)	11.0 (6.5-19.6)	7.6 (5.0-11.8)	U=57819.50	<0.001
Creatinine, mg/dL	1.00 (0.81-1.34)	1.22 (0.83-1.71)	0.99 (0.81-1.30)	U=52483.00	0.003
Lactate, mmol/L	1.56 (1.03-2.39); n=800	2.26 (1.44-3.19); n=92	1.50 (0.99-2.23); n=708	U=44091.00	<0.001
International normalized ratio	1.10 (0.99-1.30)	1.12 (0.97-1.35)	1.10 (0.99-1.29)	U=46367.00	0.487
Albumin, g/dL	3.75 (3.43-4.05); n=898	3.59 (3.25-3.85); n=97	3.77 (3.44-4.06); n=801	U=29577.50	<0.001
Shock index	0.74 (0.54-0.98)	0.93 (0.70-1.28)	0.71 (0.53-0.95)	U=60153.00	<0.001
Glasgow-Blatchford score	9.0 (4.0-12.0)	12.0 (7.8-15.0)	8.0 (4.0-12.0)	U=61897.00	<0.001

Troponin dynamics and recorded ischemic findings

Among patients classified with acute injury, median presentation, first-repeat, and late troponin concentrations were 22.9 ng/L (IQR: 14.0-41.7; n=99), 55.7 ng/L (IQR: 40.1-97.2; n=102), and 56.7 ng/L (IQR: 38.6-83.2; n=87), respectively. Among the 97 with both presentation and first-repeat results, the median signed change was 31.8 ng/L (IQR: 16.6-66.5), equivalent to a relative change of 161.2% (IQR: 68.2-281.2). The median observed peak was 74.7 ng/L (IQR: 52.2-107.2), the median peak-to-reference-limit ratio was 2.9 (IQR: 1.9-4.2), and the median observed maximum-minimum range was 49.9 ng/L (IQR: 27.6-82.5).

The acute-change rule was first met at the scheduled first-repeat sample in 90 patients (86.5%) and at the late sample in 14 (13.5%). First meeting the criterion at the late measurement does not necessarily indicate newly developing injury, because late classification may also reflect incomplete earlier sampling. The median time to first meeting the rule was 5.1 h (IQR: 4.3-5.6). The maximum observed concentration occurred at presentation in 14 (13.5%), the first repeat in 40 (38.5%), and the late draw in 50 patients (48.1%). The median time to the observed maximum was 5.8 h (IQR: 4.4-22.0). These estimates describe sampled concentrations within 24 hours and do not establish the timing of the biological peak. Table 2 summarizes all four phenotypes, and Figure 2 presents complete-case trajectories and peak distributions.

**Table 2:** Troponin kinetics by biochemical phenotype Values are presented as median (IQR), followed by the number of observed measurements or evaluable paired calculations. Concentrations are cTnI in ng/L. NA indicates no evaluable paired measurements or a measure that does not apply. The classification uses the reference limits (16 ng/L in females and 34 ng/L in males), at least two samples spanning ≥3 h, and both an absolute range ≥10 ng/L and a range ≥20% of the first observed value. Peak means maximum sampled value within 24 h. The full time-course comparison is descriptive because the grouping itself depends on troponin values and changes.

Measure	No biochemical injury detected (n=725)	Elevation without qualifying change (n=131)	Acute injury (n=104)	Indeterminate (n=40)
Presentation troponin, ng/L	8.0 (5.1-11.8); n=708	67.0 (48.9-102.9); n=129	22.9 (14.0-41.7); n=99	8.8 (5.7-16.6); n=26
First-repeat troponin, ng/L	7.9 (5.0-11.4); n=683	65.8 (47.9-105.2); n=127	55.7 (40.1-97.2); n=102	4.9 (4.2-7.1); n=3
Late troponin, ng/L	7.9 (5.1-11.5); n=578	66.5 (55.9-114.9); n=100	56.7 (38.6-83.2); n=87	6.6 (6.2-7.1); n=2
Signed presentation to first-repeat change, ng/L	0.0 (-0.4-0.4); n=666	0.0 (-1.4-1.5); n=125	31.8 (16.6-66.5); n=97	NA; n=0
Signed relative first-repeat change, %	0.6 (-5.6-5.7); n=666	0.1 (-2.1-2.3); n=125	161.2 (68.2-281.2); n=97	NA; n=0
Observed peak, ng/L	8.3 (5.3-12.2); n=725	67.0 (49.9-105.6); n=131	74.7 (52.2-107.2); n=104	8.2 (5.2-14.2); n=31
Observed peak divided by reference limit	0.3 (0.2-0.4); n=725	2.8 (2.0-3.9); n=131	2.9 (1.9-4.2); n=104	0.3 (0.2-0.5); n=31
Observed maximum minus minimum, ng/L	0.6 (0.3-1.1); n=725	2.7 (1.4-4.4); n=131	49.9 (27.6-82.5); n=104	NA; n=0
Observed sampling span, h	20.9 (19.8-22.3); n=725	20.8 (17.7-22.2); n=131	21.2 (19.9-22.0); n=104	NA; n=0
Time to observed peak, h	4.4 (0.5-20.6); n=725	4.9 (0.5-20.8); n=131	5.8 (4.4-22.0); n=104	0.6 (0.3-0.7); n=31
Time to first meeting the acute rule, h	NA; n=0	NA; n=0	5.1 (4.3-5.6); n=104	NA; n=0

**Figure 2:**
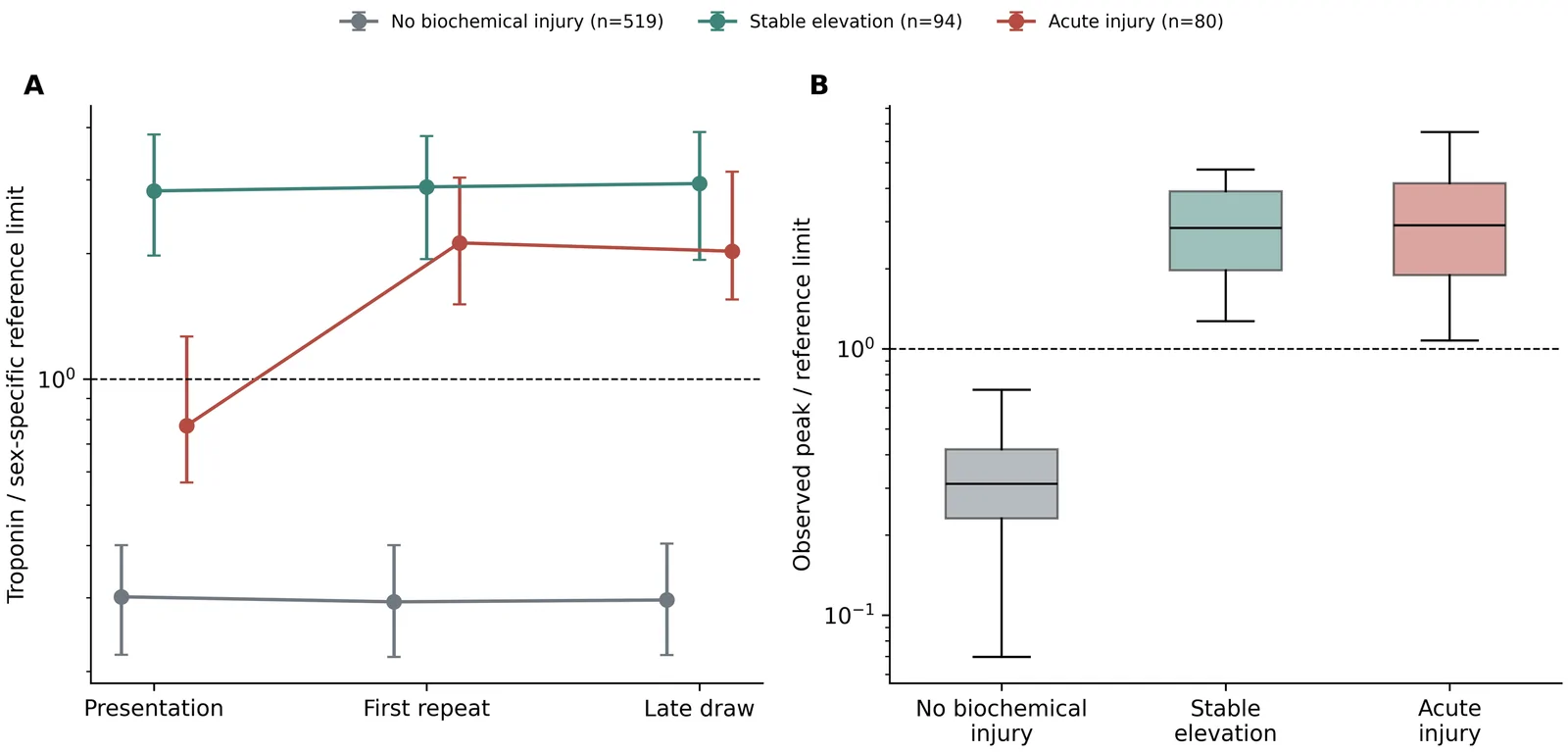
Serial troponin trajectories and observed peak concentrations Panel A shows median troponin-to-reference-limit ratios and interquartile ranges in patients with all three scheduled measurements: no biochemical injury detected (n=519), elevation without qualifying change (n=94), and acute injury (n=80). The x-axis identifies scheduled visits and does not imply equal actual sampling intervals. Panel B shows peak/reference-limit distributions for all classifiable patients in each phenotype. Boxes span the interquartile range; central lines mark medians; whiskers extend to observations within 1.5 IQR; and outliers are suppressed. Both y-axes are logarithmic. Dashed lines mark the reference limit. Elevation without qualifying change can have high concentrations despite lacking a qualifying dynamic change.

At least one recorded indicator of myocardial ischemia was present in 54 of 104 acute-injury patients (51.9%), including ischemic symptoms in 40 (38.5%) and new ischemic electrocardiogram (ECG) findings in 38 (36.5%). Echocardiography was recorded in 71 (68.3%), of whom 19 (26.8%) had a new regional wall-motion abnormality. Coronary evaluation was recorded in 17 (16.3%), with thrombus documented in five of those assessed (29.4%). Findings overlapped.

Admission factors associated with acute injury

The multivariable logistic model included 960 patients and 104 acute-injury events, with eight predictor coefficients, including center. Older age was associated with greater odds of acute injury (aOR per 10-year increase: 1.40; 95% CI: 1.21-1.63; p<0.001), as were coronary artery disease (aOR: 1.82; 95% CI: 1.06-3.10; p=0.029) and lower hemoglobin (aOR per 1 g/dL decrease: 1.27; 95% CI: 1.06-1.51; p=0.008). Sex, chronic kidney disease, systolic pressure, heart rate, and center did not show clear independent associations at the 0.05 level in this model (Table 3).

**Table 3:** Admission factors associated with acute injury Unpenalized maximum-likelihood logistic regression included 960 patients and 104 acute-injury events, with eight predictor coefficients. All listed covariates were included together in the adjusted model. Reference categories were female sex, absence of coronary artery disease, absence of chronic kidney disease, and Center 1. Acute injury was coded as the outcome event, with no acute injury as the outcome reference category. The no-acute-injury group includes elevation without qualifying change and no biochemical injury detected. ORs for continuous predictors are scaled as labelled. Center was included as a fixed effect. Confidence intervals and two-sided p-values use observed-information Wald inference. The maximum variance inflation factor was 3.26. A post hoc spline analysis detected possible nonlinearity, particularly for age. Association estimates do not constitute a validated prediction model. Center 1 denotes Gujranwala Teaching Hospital, Gujranwala, Pakistan, and Center 2 denotes Shalamar Hospital, Lahore, Pakistan.

Admission factor	Crude OR (95% CI)	Crude P	Adjusted OR (95% CI)	Wald z	Adjusted P
Age per 10 years	1.39 (1.21-1.60)	<0.001	1.40 (1.21-1.63)	4.40	<0.001
Male sex	1.49 (0.96-2.34)	0.077	1.53 (0.95-2.45)	1.75	0.079
Coronary artery disease	1.98 (1.23-3.18)	0.005	1.82 (1.06-3.10)	2.19	0.029
Chronic kidney disease	2.15 (1.30-3.54)	0.003	1.44 (0.84-2.49)	1.32	0.187
Hemoglobin per 1 g/dL decrease	1.46 (1.31-1.63)	<0.001	1.27 (1.06-1.51)	2.65	0.008
Systolic pressure per 10 mmHg decrease	1.32 (1.19-1.45)	<0.001	1.10 (0.95-1.28)	1.26	0.206
Heart rate per 10 beats/min	1.33 (1.22-1.46)	<0.001	1.11 (0.95-1.31)	1.33	0.185
Center 2 versus Center 1	1.06 (0.71-1.60)	0.771	0.98 (0.63-1.52)	-0.09	0.932

The maximum variance inflation factor was 3.26. Exploratory spline analysis suggested departure from the assumption of linear continuous effects (likelihood-ratio χ²=11.22; 4 degrees of freedom; p=0.024), including possible age nonlinearity (p=0.031). Accordingly, the age odds ratio per decade summarizes the fitted linear model and should not be interpreted as a constant association across all ages. Overall associations for age and hemoglobin remained evident in the exploratory spline model (p<0.001 and p=0.018, respectively).

Full-admission outcomes

Among classifiable patients with known vital status, in-hospital mortality was 19 of 101 (18.8%) in the acute-injury group and 26 of 826 (3.1%) in the nonacute group (unadjusted risk ratio (RR): 5.98; 95% CI: 3.43-10.40; Fisher exact p<0.001). Acute injury was also associated with greater observed risks of ICU admission (31.7% vs. 14.8%; RR: 2.14; 95% CI: 1.55-2.96; p<0.001), rebleeding (11.5% vs. 5.8%; RR: 1.98; 95% CI: 1.09-3.59; p=0.026), invasive ventilation (10.6% vs. 4.3%; RR: 2.45; 95% CI: 1.29-4.65; p=0.006), and vasopressor use (10.6% vs. 4.6%; RR: 2.32; 95% CI: 1.23-4.39; p=0.009).

Red-cell transfusion was recorded in 55.8% versus 30% of patients (RR: 1.86; 95% CI: 1.52-2.27; p<0.001), with median requirements of 1.0 (IQR: 0.0-3.0) and 0.0 units (IQR: 0.0-1.0), respectively. Among patients undergoing endoscopy, the median time to the procedure was longer with acute injury (14.4 vs. 12.1 h; p=0.004). The median observed index-care length of stay was 4.5 versus 3.3 days (p<0.001). Endoscopy and endoscopic-hemostasis rates were similar between groups. The association with recorded acute kidney injury was uncertain (13.5% vs. 8.1%; RR: 1.67; 95% CI: 0.98-2.86; p=0.064). These full-admission comparisons are descriptive because some interventions or outcomes may have occurred before injury ascertainment was complete (Table 4).

**Table 4:** Full admission outcomes by acute injury status Categorical values are presented as n/N (%); continuous values are presented as median (IQR). RR confidence intervals use the logarithmic Katz method. Mortality was compared using a two-sided Fisher exact test; other binary outcomes used Pearson chi-squared tests without continuity correction, with 1 degree of freedom. Continuous outcomes used two-sided Mann-Whitney U tests, with U reported for the acute-injury group. Mortality is restricted to known outcomes (n=101 and n=826); three and 30 transfers, respectively, have unknown mortality. Other binary outcomes describe events documented during observed index care, including care before transfer; they do not establish complete post-transfer ascertainment. Endoscopy timing is restricted to patients undergoing endoscopy (n=94 and n=782). Length of stay ends at death, discharge, or transfer. Full-admission associations are descriptive because some events may have preceded completion of injury ascertainment. P-values for secondary outcomes were not adjusted for multiple comparisons and are intended to generate hypotheses; apparently significant findings may therefore include false-positive associations.

Outcome	Acute injury (n=104)	No acute injury (n=856)	Unadjusted RR (95% CI)	Test statistic	P-value
In-hospital mortality	19/101 (18.8%)	26/826 (3.1%)	5.98 (3.43-10.40)	Fisher exact	<0.001
ICU admission	33/104 (31.7%)	127/856 (14.8%)	2.14 (1.55-2.96)	χ²=19.06	<0.001
Rebleeding	12/104 (11.5%)	50/856 (5.8%)	1.98 (1.09-3.59)	χ²=4.98	0.026
Recorded acute kidney injury	14/104 (13.5%)	69/856 (8.1%)	1.67 (0.98-2.86)	χ²=3.42	0.064
Invasive ventilation	11/104 (10.6%)	37/856 (4.3%)	2.45 (1.29-4.65)	χ²=7.64	0.006
Vasopressor use	11/104 (10.6%)	39/856 (4.6%)	2.32 (1.23-4.39)	χ²=6.81	0.009
Endoscopy performed	94/104 (90.4%)	782/856 (91.4%)	0.99 (0.93-1.06)	χ²=0.11	0.741
Endoscopic hemostasis	35/104 (33.7%)	338/856 (39.5%)	0.85 (0.64-1.13)	χ²=1.33	0.249
Any red-cell transfusion	58/104 (55.8%)	257/856 (30%)	1.86 (1.52-2.27)	χ²=27.88	<0.001
Red-cell units transfused	1.0 (0.0-3.0)	0.0 (0.0-1.0)	Not applicable	U=57703.00	<0.001
Time to endoscopy, h	14.4 (10.2-19.6)	12.1 (8.6-16.7)	Not applicable	U=43360.00	0.004
Observed index-care length of stay, days	4.5 (3.4-6.0)	3.3 (2.2-4.6)	Not applicable	U=58764.00	<0.001

Landmark mortality and sensitivity analyses

Before or at the 24-hour landmark, 143 patients had exited index care: 124 were discharged alive, 14 died, and five were transferred. Among the 857 still in index care, 19 had indeterminate injury status, leaving 838 landmark-eligible patients: 95 with acute injury and 743 without acute injury. Subsequent mortality remained unknown in three and 25 patients, respectively. The principal mortality model therefore included 810 patients with known outcomes and 34 deaths.

Subsequent mortality was 14 of 92 (15.2%) with acute injury and 20 of 718 (2.8%) without acute injury (unadjusted RR: 5.46; 95% CI: 2.86-10.44). The absolute risk difference was 12.4 percentage points (95% CI: 6.3-21.2). Acute injury remained associated with subsequent mortality after adjustment for age, GBS, and chronic kidney disease (aOR: 3.47; 95% CI: 1.58-7.62; p=0.002). Estimates were similar in the reduced model (aOR: 3.41; 95% CI: 1.57-7.42), after additional adjustment for center (aOR: 3.53; 95% CI: 1.60-7.80), and with Firth bias reduction (point estimate: 3.45). The primary model had 8.5 deaths per predictor coefficient, so precision remained limited.

Restriction to patients completing all three measurements yielded an acute-injury prevalence of 11.5% (80/693; 95% CI: 9.4-14.1%). In the corresponding landmark mortality analysis, the aOR was 3.25 (95% CI: 1.42-7.44; p=0.005). Excluding elevation without qualifying change from the comparator yielded an aOR of 3.39 (95% CI: 1.48-7.78; p=0.004). Prevalence was 18.6% in patients with chronic kidney disease versus 9.6% without it and 12.9% in variceal versus 10.3% in confirmed nonvariceal bleeding (Table 5).

**Table 5:** Prevalence sensitivity analyses and subgroups Prevalence is the number of patients with acute injury divided by the number with classifiable assessments in each subgroup; 95% CIs were calculated using the Wilson method. Indeterminate assessments are listed separately and excluded from prevalence denominators. Confirmed nonvariceal bleeding includes peptic ulcer disease, erosive disease, Mallory-Weiss lesions, malignancy, and other confirmed upper gastrointestinal sources; unconfirmed sources are kept separate. Subgroup estimates are descriptive, and the study was not powered for interaction testing. Center 1 denotes Gujranwala Teaching Hospital, Gujranwala, Pakistan, and Center 2 denotes Shalamar Hospital, Lahore, Pakistan.

Population	Acute/classifiable	Prevalence % (95% CI)	Indeterminate n
All classifiable assessments	104/960	10.8 (9.0-13.0)	40
All three measurements observed	80/693	11.5 (9.4-14.1)	0
No chronic kidney disease	80/831	9.6 (7.8-11.8)	35
Chronic kidney disease	24/129	18.6 (12.8-26.2)	5
Variceal bleeding	19/147	12.9 (8.4-19.3)	1
Confirmed nonvariceal bleeding	75/729	10.3 (8.3-12.7)	29
Unconfirmed bleeding source	10/84	11.9 (6.6-20.5)	10
Center code 1	57/539	10.6 (8.3-13.5)	21
Center code 2	47/421	11.2 (8.5-14.5)	19
Female	30/353	8.5 (6.0-11.9)	17
Male	74/607	12.2 (9.8-15.0)	23

Assigning all unresolved landmark mortality outcomes to survival or death yielded aORs of 3.44 and 2.39, respectively. In an adverse differential scenario assigning all three unresolved acute-injury outcomes to survival and all 25 unresolved nonacute outcomes to death, the aOR decreased to 1.86 (95% CI: 0.94-3.69; p=0.074). Thus, the mortality association was sensitive to sufficiently adverse assumptions about missing outcomes. The model and sensitivity estimates are presented in Table 6. 

**Table 6:** Landmark mortality and sensitivity models Adjusted odds ratios were estimated using multivariable binary logistic regression fitted by unpenalized maximum likelihood. The displayed 95% CIs and two-sided p-values were calculated using Wald inference. The outcome was subsequent in-hospital death, and the exposure comparison was acute injury versus no acute injury. The primary model adjusts for age per 10 years, Glasgow-Blatchford score per point, and chronic kidney disease. The reduced model omits chronic kidney disease, and the model plus center additionally includes Center 2 versus Center 1 as a fixed effect. Eligibility requires survival and continued index care beyond 24 hours and classifiable injury status. The primary model excludes 28 unresolved subsequent outcomes (three acute injury and 25 no acute injury). The adverse differential scenario assigns the three unresolved acute-injury outcomes to survival and the 25 unresolved no-acute-injury outcomes to death; the opposite scenario reverses these assignments. These extreme scenarios are not imputations used in the primary analysis. Firth bias-reduced logistic regression gave an injury OR of 3.45; a profile-likelihood confidence interval was not calculated, so this separate sensitivity result is presented as a point estimate only.

Analysis	Analyzed n	Deaths	Acute injury aOR (95% CI)	P-value
Primary landmark model	810	34	3.47 (1.58-7.62)	0.002
Reduced landmark model	810	34	3.41 (1.57-7.42)	0.002
Landmark model plus center	810	34	3.53 (1.60-7.80)	0.002
Complete three-draw landmark patients	659	32	3.25 (1.42-7.44)	0.005
Acute versus no biochemical injury	701	31	3.39 (1.48-7.78)	0.004
Missing landmark mortality assigned survival	838	34	3.44 (1.57-7.51)	0.002
Missing landmark mortality assigned death	838	62	2.39 (1.26-4.55)	0.008
Adverse differential missing-outcome scenario	838	59	1.86 (0.94-3.69)	0.074
Opposite differential missing-outcome scenario	838	37	4.57 (2.18-9.61)	<0.001

## Discussion

Principal findings and comparison with previous studies

In this multicenter cohort of adults presenting with UGIB, acute myocardial injury was identified in 10.8% of patients with classifiable serial assessments. Older age, coronary artery disease, and lower admission hemoglobin were associated with acute injury in the adjusted analysis. Patients with acute injury also experienced greater observed mortality, organ-support requirements, transfusion exposure, and length of stay. Among patients alive and still hospitalized at 24 hours, acute injury remained associated with subsequent in-hospital mortality after adjustment for age, GBS, and chronic kidney disease. These findings suggest that acute myocardial injury identifies a subgroup with a more complicated clinical course, although they do not establish that myocardial injury causes these outcomes or that troponin-guided management improves prognosis.

Previous studies have reported heterogeneous frequencies of troponin elevation during gastrointestinal bleeding. Iser et al. detected troponin I above their study threshold in 19% of 156 patients with UGIB using measurements at presentation, 12 hours, and 24 hours [4]. Iqbal et al. reported elevation in 10% of 290 patients with acute gastrointestinal bleeding, whereas Kousa et al. found abnormal troponin in 17% of 172 tested patients [5,6]. These estimates are not directly interchangeable with the present acute-injury prevalence. The earlier studies differed in bleeding populations, selection for troponin testing, assay thresholds, and requirements for dynamic change. In the present study, acute injury and elevation without qualifying change together accounted for 235 of 960 classifiable assessments (24.5%). Thus, an estimate based on any elevation would describe a different population from the 10.8% meeting the acute-change criterion.

Ullah et al. reported myocardial injury in 8% of 163 patients with UGIB in Peshawar, excluding patients with pre-existing cardiac disease and using a troponin I threshold above 0.2 ng/mL [7]. Their study included two measurements 12 hours apart, but its reported definition did not require the combined absolute and relative changes applied here. Inclusion of patients with cardiovascular and renal disease, together with different reference limits and classification rules, may partly explain the differing estimates. These methodological differences prevent attributing the variation to geography or a greater underlying incidence. The present study therefore extends earlier serial-testing work through explicit separation of biochemical phenotypes and a temporally defined mortality analysis, rather than introducing serial testing itself.

Interpretation of myocardial injury and associated admission factors

The distinction between acute injury and elevation without qualifying change is central to interpreting these findings. Elevation without qualifying change was identified in 13.6% of classifiable patients, and its median observed peak concentration approached that of the acute-injury group. A relatively high concentration therefore did not necessarily indicate a qualifying acute change. Contemporary troponin interpretation requires attention to serial measurements, assay-specific reference limits, and clinical context [10,11]. Conversely, stability during a limited 24-hour window cannot definitively establish that an elevation is chronic: an earlier injury may already have reached a plateau before presentation. The study categories should consequently be understood as observed biochemical phenotypes rather than definitive descriptions of the timing or cause of myocardial damage.

The associations with age, coronary artery disease, and hemoglobin are compatible with increased myocardial vulnerability during hemorrhagic illness. Anemia reduces arterial oxygen-carrying capacity, while hypovolemia, hypotension, and tachycardia may further disturb the balance between myocardial oxygen delivery and demand [10,12]. Against this physiological background, established coronary disease and reduced cardiovascular reserve offer plausible explanations for the observed associations with age and coronary history. These mechanisms were not directly measured, however, and cannot be assigned to individual patients from the regression results. In particular, the hemoglobin association does not establish a transfusion threshold or demonstrate that correcting anemia beyond standard indications would prevent myocardial injury.

Lower systolic pressure and higher heart rate were associated with injury in unadjusted comparisons but did not show clear independent associations in the full model. Their attenuation may reflect overlapping information about bleeding severity, relationships with hemoglobin and comorbidity, or limited precision. It should not be interpreted as evidence that hemodynamic instability is clinically unimportant. Similarly, acute injury was more frequent in patients with chronic kidney disease, but the adjusted estimate remained uncertain. The lack of a statistically clear adjusted association does not exclude an effect of renal disease or justify dismissing dynamic troponin changes in these patients. The age association also requires qualification: exploratory spline analyses suggested nonlinearity, so the reported odds ratio per decade represents a summary of the prespecified linear model rather than a constant effect across all ages.

Recorded ischemic findings further demonstrate the heterogeneity of acute injury. At least one ischemic indicator was documented in 51.9% of patients classified with acute injury, but echocardiography and coronary evaluation were performed selectively. The absence of a recorded abnormality therefore cannot be equated with a negative comprehensive cardiac assessment. Myocardial infarction requires evidence of ischemia in addition to acute myocardial injury, and a diagnosis of type 2 infarction cannot be inferred from anemia or bleeding alone [10]. Consequently, these results should not be interpreted as an infarction prevalence estimate. Mechanistic classification remains limited by the availability of clinical information and cardiac investigations.

Troponin dynamics and clinical outcomes

The serial measurements illustrate why a single presentation concentration may incompletely characterize injury during UGIB. Most acute-injury cases first met the classification rule at the first repeat, but 13.5% did so only at the late measurement. Moreover, almost half of the acute-injury group had their maximum observed concentration at the late draw. These findings support further evaluation of serial sampling strategies while not establishing that every patient requires the same three-sample schedule. Time to first meeting the rule is partly determined by sampling intervals, and the sampled maximum is not necessarily the biological peak. Because the phenotype definition itself incorporates troponin change, the contrasting trajectories are descriptive features of the classification rather than independent validation of its diagnostic accuracy.

The mortality findings are directionally consistent with those of Iqbal et al., who reported higher 30-day mortality among patients with elevated troponin, but differ from Kousa et al., who found no statistically significant mortality difference [5,6]. Differences in selection, injury definitions, case mix, and follow-up may contribute to this inconsistency. The current landmark analysis adds temporal separation by restricting injury ascertainment to the first 24 hours and evaluating subsequent deaths among patients still in care. The adjusted odds ratio of 3.47 indicates an association within this selected population; it should not be interpreted as a risk ratio, and its interpretation does not extend to patients who died or left care before the landmark. Persistence after GBS adjustment also does not establish incremental predictive performance beyond that score.

The mortality association was similar across several alternative models, including restriction to complete sampling and exclusion of elevation without qualifying change from the comparator. Nevertheless, unresolved outcomes remain consequential. In the adverse differential scenario, the adjusted estimate decreased to 1.86, and its confidence interval included the null. This scenario represents an extreme assignment rather than an estimate of what actually happened, but it shows that the result is not insensitive to missing follow-up. The results therefore support an association with higher observed subsequent mortality, subject to uncertainty from residual confounding, limited event numbers, and incomplete outcome ascertainment.

The associations with ICU admission, ventilation, vasopressor use, rebleeding, and transfusion indicate a greater observed burden of illness. Some of these events may have preceded completion of injury classification, however, and may represent causes, consequences, or parallel manifestations of physiological deterioration. Longer time to endoscopy and hospital stay resemble the resource-use findings reported by Kousa et al. [6]. In this cohort, the reasons for endoscopy timing were not established; resuscitation needs, clinical decision-making, or service availability could all contribute. It would therefore be inappropriate to conclude that cardiac investigation caused the delay or that the modest timing difference explains the mortality association. Likewise, length of stay is affected by death and transfer and should not be treated as an uncomplicated measure of recovery.

Clinical implications

These findings support interpreting troponin results as part of an integrated assessment of patients with UGIB. When acute injury is suspected, serial concentrations, symptoms, ECG findings, and relevant cardiac imaging can help clarify its significance [10,11]. Management must also address the bleeding episode and physiological instability; evaluation of possible cardiac injury should be coordinated with appropriately timed hemostatic care [2]. An elevated troponin concentration alone is insufficient justification for a type 1 acute coronary syndrome treatment pathway. Decisions about antithrombotic therapy or invasive coronary evaluation require assessment of the likelihood of an ischemic coronary event against the risks of active bleeding [12].

The study does not establish the benefit, safety, or cost-effectiveness of routine troponin screening in all patients with UGIB. Nor does it show that more intensive monitoring, different transfusion targets, or a specific endoscopy schedule improves outcomes in patients with acute injury. The observed admission associations may help define groups for future evaluation, but they should not be converted into a clinical prediction rule without validation. In particular, a statistically significant adjusted troponin coefficient does not demonstrate improved discrimination, calibration, reclassification, or clinical usefulness when troponin is added to existing bleeding-risk assessment.

Strengths and limitations

The study's strengths include its prospective multicenter design, the relatively large number of enrolled patients, and planned troponin assessment across defined early sampling windows. Patients with established cardiovascular or renal disease were retained, allowing examination of clinically relevant comorbidity rather than restricting the cohort to patients without these conditions. Explicit distinction between acute injury, elevation without qualifying change, and indeterminate status reduced the risk of treating every elevation as acute or every incomplete assessment as negative. The landmark analysis, reporting of available denominators, and sensitivity analyses also make the temporal and missing-data assumptions more transparent.

This study has several limitations. First, its prospective observational cohort design involved the systematic collection of clinical and laboratory data and follow-up of patients without investigator-assigned interventions or manipulation of clinical exposures. Although this approach enabled the assessment of associations between acute myocardial injury and subsequent clinical outcomes under routine practice conditions, it does not establish a causal relationship. Unlike randomized experimental studies, observational designs remain susceptible to confounding because patients with and without myocardial injury may differ in underlying cardiovascular disease, bleeding-related clinical risk, concurrent illnesses, and treatment-related factors. Although multivariable regression was employed to adjust for selected measured characteristics, residual confounding from unmeasured or incompletely captured variables cannot be excluded. Although injury status was classifiable in 96% of participants, only 69.3% completed all three scheduled measurements. Patients without late sampling could have developed qualifying injury after their final observed sample, resulting in underascertainment. The combined absolute and relative change rule was prespecified but has not been validated specifically in UGIB. Elevation without qualifying change does not establish chronic injury, while the absence of elevation on available samples does not exclude injury outside the observed sampling period. Selective cardiac imaging and coronary assessment limited interpretation of injury mechanisms [10,11]. The landmark mortality model included only 34 deaths, limiting precision and the extent of reliable adjustment. Possible age nonlinearity also limits interpretation of a constant odds ratio per decade. The landmark analysis applies only to patients surviving and remaining hospitalized at 24 hours. Unresolved outcomes after transfer materially affected the mortality sensitivity analyses. Cause-specific mortality, recent myocardial infarction or cardiac procedures, and long-term outcomes were unavailable. Finally, recruitment from two tertiary centers and multiple exploratory comparisons limit generalizability and increase uncertainty.

Future studies should combine verified assay-specific protocols with more complete serial sampling, documented clinical adjudication, and follow-up after transfer and discharge. External cohorts are needed to assess whether early myocardial injury adds useful prognostic information beyond established bleeding scores and whether its associations differ across renal disease, bleeding source, and ischemic phenotypes. Only after establishing reproducible classification and incremental clinical usefulness should intervention studies determine whether a troponin-informed care strategy improves outcomes without increasing bleeding-related harm.

## Conclusions

Acute myocardial injury, defined using the prespecified study-specific troponin criterion, was detected in 10.8% of patients with classifiable assessments. Older age, coronary artery disease, and lower admission hemoglobin were associated with injury. Among patients alive and still hospitalized at 24 hours with known subsequent outcomes, acute injury was associated with higher in-hospital mortality. This mortality association was observed in a selected landmark population comprising patients who remained alive and hospitalized at 24 hours, and its magnitude was attenuated under an adverse missing-outcome sensitivity scenario. Consequently, these findings should not be interpreted as evidence of causality or as demonstrating incremental prognostic utility beyond established UGIB risk stratification tools. Incomplete sampling, limited mortality events, unresolved outcomes, and residual confounding warrant cautious interpretation. Serial measurements distinguished qualifying dynamic changes from elevation without qualifying change, but these biochemical categories do not establish the underlying mechanism or demonstrate that troponin-guided management improves outcomes.
